# *Schaeferiana* (*Gaboniella* subgen. n.) *incompleta* sp. n. from Gabon, with notes on its relationships  and new records from the Central African Republic (Hemiptera, Heteroptera, Pyrrhocoridae)

**DOI:** 10.3897/zookeys.126.1723

**Published:** 2011-09-02

**Authors:** Jaroslav L. Stehlík, Zdeněk Jindra

**Affiliations:** 1 Department of Entomology, Moravian Museum, Hviezdoslavova 29a, CZ-627 00 Brno – Slatina, Czech Republic; 2 Department of Plant Protection, Faculty of Agrobiology, Food and Natural Resources, Czech University of Agriculture, CZ-165 21 Praha 6-Suchdol, Czech Republic

**Keywords:** Heteroptera, Pyrrhocoridae, taxonomy, new species, Gabon, Central African Republic, Afrotropical Region

## Abstract

A new subgenus, *Gaboniella* **subgen. n.**, of the genus *Schaeferiana* Stehlík, 2008, and its type species, *Schaeferiana (Gaboniella) incompleta* **sp. n.** are described from Gabon. In addition the first state records of *Sericocoris (Depressoculus) albomaculatus* Stehlík, 2008 and *Schaeferiana (Schaeferiana) mirabilis* Stehlík, 2008 from the Central African Republic are provided.

## Introduction

In this contribution, we describe the subgenus, *Gaboniella* subgen. n., of the genus *Schaeferiana* Stehlík, 2008, and its type species, *Schaeferiana (Gaboniella) incompleta* sp. n. from Gabon. This species is interesting from morphological and phylogenetic point of view, as it possesses characters placing it between the subgenus *Depressoculus* Stehlík, 2008 of *Sericocoris* Karsch, 1892 and the genus *Schaeferiana* Stehlík, 2008, especially in the peculiar structure of the head and pronotum. As the new species does not fit the diagnosis of the genus *Schaeferiana* in some points, we establish a new subgenus to accommodate it.

*Sericocoris (Depressoculus)* includes two species distributed in tropical Africa from Liberia to Democratic Republic of Congo: *Sericocoris (Depressoculus) albomaculatus* Stehlík, 2008 (western, north-western and eastern parts of the Democratic Republic of Congo – provinces Kwilu, Sud-Kivu, Nord-Kivu, Nord-Ubangi and Sud-Ubangi) and the widely distributed *Sericocoris (Depressoculus) antennatus* (Distant, 1881), which includes four described subspecies: *Sericocoris (Depressoculus) antennatus antennatus* (Liberia, Nigeria, Cameroon, Equatorial Guinea: Bioko Island, Gabon, Congo), *Sericocoris (Depressoculus) antennatus obscuratus* Stehlík, 2008 (western, north-western, and north-central parts of the Democratic Republic of Congo – provinces Bas-Uelé, Equateur, Kwilu, Mongala, Sud-Ubangi, Tshopo and Tsuapa), *Sericocoris (Depressoculus) antennatus immaculatus* Stehlík, 2008 (north-eastern and eastern areas of the Democratic Republic of Congo – Bas-Uelé, Haut-Uelé, Ituri, Nord-Kivu, and Sud-Kivu), and *Sericocoris (Depressoculus) antennatus congolanus* Stehlík, 2008 (south-west of the Democratic Republic of Congo – province Kongo Central) (Stehlík 2008). The genus *Schaeferiana* was so far monotypical, including only *Sericocoris mirabilis* Stehlík, 2008 from north-western, north-central, and eastern areas of the Democratic Republic of Congo (Maniema, Nord-Kivu, Sud-Kivu, and Tshopo provinces) (Stehlík 2008). The present records of *Sericocoris (Depressoculus) albomaculatus* and *Sericocoris mirabilis* from the Central African Republic shift the distribution of these species further west.

## Materials and methods

To a large extent, the terminology for body parts in this contribution follows van Doesburg (1968), but the more specific terms proposed by Schaefer (1977) are employed for the genital capsule.

## Taxonomy

### Schaeferiana

Stehlík, 2008

http://species-id.net/wiki/Schaeferiana

Schaeferiana  Stehlík, 2008: 14–19.

#### Type species:

 *Schaeferiana mirabilis* Stehlík, 2008, by original designation.

#### Diagnosis.

Pronotal collar widened. Anterior, pale portion of pronotum elevated.

#### Discussion.

 Stehlík (2008) established *Schaeferiana* as a new genus with particular reference to its structure of the pronotal collar and callar lobe, which is unique among all Pyrrhocoridae:

‘Pronotum rather long, widening markedly towards base, posterior angles and posterior margin distinctly rounded; lateral margins strongly raised dorsally, concave (deeply in males, slightly in females); at level of callar lobe lateral margins wider than anteriorly. Pronotal collar projecting posteriad, forming rather large, horizontal plate of rectangular shape with rounded angles and irregular shallow imprints, extended above callar lobe. Base of callar lobe rudimentary, its anterior two-thirds cavernous, mostly covered by the rectangular projection of the pronotal collar, visible only as a narrow fissure. Anterolateral margin of the rudimentary part usually extended above level of the median part and strongly convex. Pronotal lobe towards base uniformly gibbous, anterolaterally deeply depressed’ (Stehlík 2008). However, based on the new material available, this original diagnosis must be modified – the pale, transverse, ridge-shaped structure interpreted by Stehlík (2008) as posterior third of callar lobe, in fact, represents the anterior margin of the pronotal lobe.

However, *Schaeferiana (Gaboniella) incompleta* sp. n. does not fit the diagnosis of the genus *Schaeferiana* in some points, so we establish a new subgenus to accommodate it. This species is interesting from morphological and phylogenetic point of view, as it possesses characters placing it between the subgenus *Depressoculus* of *Sericocoris* and the genus *Schaeferiana*, especially in the structure of head and pronotum.

#### Key to the subgenera of Schaeferiana Stehlík, 2008

**Table:** 

1(2)	Pronotal collar slightly widened (length 0.27 mm (♂)), touching tightly the slightly gibbose callar lobe of pronotum. Anterior, pale portion of pronotal lobe only slightly elevated above the surrounding surface (not reaching the lateral margins). Head dorsally without wrinkles or a median furrow.	*Gaboniella* subgen. n.
2(1)	Pronotal collar strongly widened (length 0.59 mm (♂)), in the form of a horizontal plate covering the strongly depressed callar lobe posteriorly, so there is a nearly closed space except for a narrow slit posteriorly. Anterior, pale portion of pronotal lobe strongly gibbose, ridge-like. Head dorsally wrinkled with developed median furrow.	*Schaeferiana* s. str.

### Gaboniella subgen. n.

urn:lsid:zoobank.org:act:805012F6-8CEE-411B-A12B-52705DC8F033

http://species-id.net/wiki/Schaeferiana_(Gaboniella)

#### Type species.

*Schaeferiana (Gaboniella) incompleta* sp. n., here designated.

#### Diagnosis.

Differing from the nominotypical subgenus in the characters given in the key.

#### Etymology.

The name of the subgenus is formed from the area of origin, Gabon; gender feminine.

### Schaeferiana (Gaboniella) incompleta sp. n.

urn:lsid:zoobank.org:act:94C280F9-3F7E-4F73-9357-8499C471A1BD

http://species-id.net/wiki/Schaeferiana_(Gaboniella)_incompleta

Figs 1 3 

#### Type material.

Holotype: ♂, **GABON:** ‘Mission biologique au Gabon, P.P. Grasse Directeur, Belinga 222, 19.iii.[19]63, H. Coiffait’ (coll. Muséum national d’Histoire naturelle, Paris).

#### Description.

**Figures 1–2.**
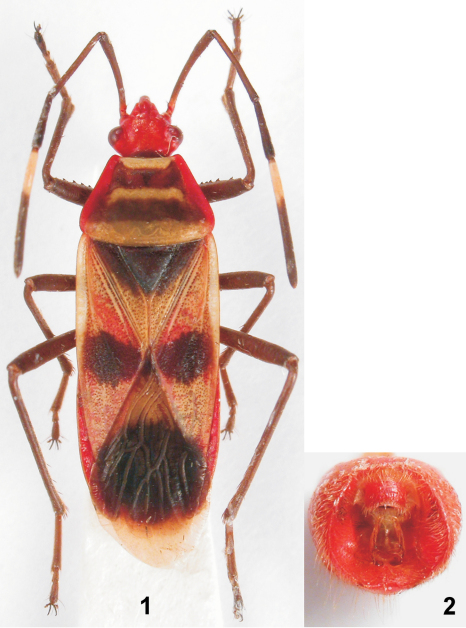
*Schaeferiana (Gaboniella) incompleta* sp. n., holotype, male. **1** habitus **2** pygophore in dorsal view.

Colouration (Fig. 1). Head both dorsally and ventrally, lateral pronotal margins, pronotal epipleuron, dorsal margin of pleural flange I, ventrites (except white stripes on posterior margins of ventrites II–V), ventral and dorsal laterotergites, and pygophore red. Antennae (except of basal third of antennomere 4), callar lobe (with reddish tinge), transverse median stripe on pronotal lobe, scutellum, base of clavus (narrowing towards apex of scutellum), median round spot on corium, apex of corium, small spot on base of membrane, large round median spot on membrane, labium, pleura I–III, and legs, including coxae and trochanters, black. Basal third of antennomere 4, widened pronotal and prosternal collar, pleural flange I (except dorsal margin), entire pleural flanges I and II, epicoxal lobes I–III, and wide stripes on posterior margins of ventrites II–V white. Slightly elevated transverse stripe anteriorly on pronotal lobe, posterior margin of pronotum (widely), apical portion of clavus, and most of corium whitish-orange. Membrane pale gray.

Structure**.**Head. Body smaller, nearly parallel-sided. Head dorsally without wrinkles, median furrow not developed. Gena under the eye with distinct rounded depression anterior to bulges, extending to the median part of temple and eye. Eyes relatively small, weakly protruding. Eye and temple dorsoventrally flattened; gena under eye with distinct depression. Labium reaching base of ventrite IV.

Pronotum short. Widened pronotal collar tightly touching the slightly gibbose callar lobe. Lateral pronotal margin wide, strongly elevated dorsally, slightly concave medially. Anterior portion of pronotal lobe very slightly elevated above surrounding surface, not reaching lateral pronotal margins. Posterior pronotal margin rounded.

Scutellum. Posterior two thirds of scutellum slightly convex.

Legs slender, long. Profemur not markedly thickend compared with meso- and metafemur, slightly attenuated basally and towards apex. Ventral face of profemur with five remote teeth. Tibiae distally with slender semi-erect spines.

Pygophore (Fig. 2). Ventral portion of ventral wall receding in lateral view. Ventral rim medially concave, with a small tooth near each side of the incision. Lateral rim elevated above the ventral rim, sharp, somewhat lower near the dorsal rim. Ventral and lateral rim infolding steeply sloping into genital chamber; lateral rim infolding with a convex at midlength, and with a larger patch of minute black denticles near the dorsal rim.

**Figure 3.**
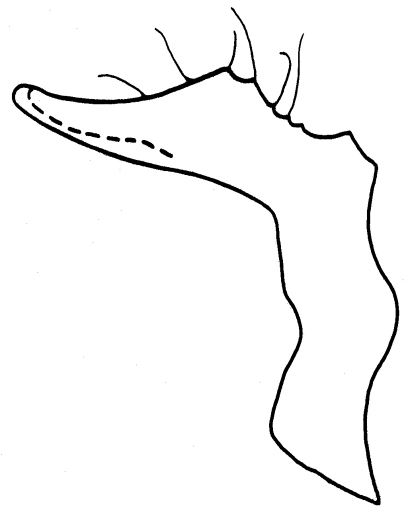
*Schaeferiana (Gaboniella) incompleta* sp. n., holotype, male, paramere.

Parameres parallel, their apices reaching up to anal tube. Paramere (Fig. 3) narrow at base, then strongly widened towards genital chamber (seen *in situ*). Dorsal margin of paramere proximally roundly incised, the incision terminated by a spine; distal part of the dorsal margin slightly concave; apex of paramere narrowly rounded.

Measurements (mm). Holotype (♂). Body length 10.8 mm; head: width (including eyes) 1.73, interocular width 1.03; lengths of antennomeres: 1 – 2.21, 2 – 1.78, 3 – 1.35, 4 – 5.43; pronotum: length 1.84, maximum width 2.97; length of pronotal collar 0.27; scutellum: length 1.40, width 1.51; corium: length 5.56, width 1.51.

#### Differential diagnosis.

 The new species differs from *Schaeferiana (Schaeferiana) mirabilis* in the characters defining both subgenera. Moreover, *Schaeferiana (Gaboniella) incompleta* is smaller, its pronotum is shorter, the lateral pronotal margins are red (black in *Schaeferiana (Schaeferiana) mirabilis*), and the membrane is pale gray with a large central black spot (mostly black with orange median spot and pale gray apical margin in *Schaeferiana (Schaeferiana) mirabilis*).

#### Etymology.

 The species epithet is the Latin adjective *incompletus*, -*a*, -*um*, meaning incomplete, referring to the incompletely modified pronotal collar and callar lobe compared with *Schaeferiana (Schaeferiana) mirabilis*.

#### Distribution.

 Known only from the type locality in Gabon.

## Faunistics

### Schaeferiana (Schaeferiana) mirabilis

Stehlík, 2008

http://species-id.net/wiki/Schaeferiana_(Schaeferiana)_mirabilis

Fig. 4 

#### Material examined.

 **Central African Republic:** WSW, Sangha province, 45 km E of Nola, (GPS) 03°40'N, 16°26'E, 570 m a.s.l., 17.xii.2008, 2 ♂♂ 1 ♀,J. Halada lgt. (coll. Z. Jindra, Praha).

#### Distribution.

Democratic Republic of Congo (Stehlík 2008). New record for the Central African Republic.

**Figures 4–5.**
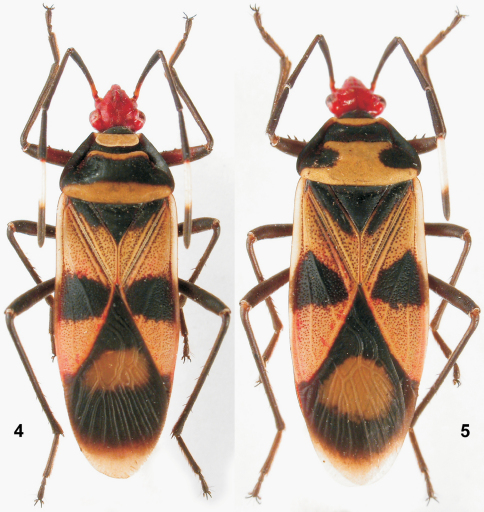
Habitus. **4** *Schaeferiana (Schaeferiana) mirabilis* Stehlík, 2008, male **5** *Sericocoris (Depressoculus) albomaculatus* Stehlík, 2008, female.

### Sericocoris (Depressoculus) albomaculatus

Stehlík, 2008

http://species-id.net/wiki/Sericocoris_(Depressoculus)_albomaculatus

Fig. 5 

#### Material examined.

 **Central African Republic:** WSW, Sangha province, 45 km E of Nola, (GPS) 03°40'N, 16°26'E, 570 m a.s.l., 17.xii.2008, 1 ♀,J. Halada lgt. (coll. Z. Jindra, Praha).

#### Distribution.

Democratic Republic of Congo (Stehlík 2008). New record for the Central African Republic.

## Supplementary Material

XML Treatment for Schaeferiana

XML Treatment for Gaboniella

XML Treatment for Schaeferiana (Gaboniella) incompleta

XML Treatment for Schaeferiana (Schaeferiana) mirabilis

XML Treatment for Sericocoris (Depressoculus) albomaculatus

## References

[B1] Doesburg PH van (1968) A revision of the New World species of *Dysdercus* Guérin Méneville (Heteroptera, Pyrrhocoridae). Zoologische Verhandelingen (Leiden) 97: 1-215

[B2] Schaefer CW (1977) Genital capsule of the trichophoran male (Hemiptera: Heteroptera: Geocorisae). International Journal of Insect Morphology and Embryology 6: 277-301 doi: 10.1016/0020-7322(77)90022-8

[B3] Stehlík JL (2008) New taxa of Afrotropical Pyrrhocoridae (Hemiptera: Heteroptera). Entomologica Basiliensia et Collectionis Frey 30: 3-20

